# Pressure-induced mechanically, thermodynamically and structurally stable half-metallic ferromagnetic NiSc_2_X_4_ (X = S and Se) spinels for spintronic applications: DFT-calculations

**DOI:** 10.1039/d6ra05435h

**Published:** 2026-08-05

**Authors:** M. Aslam Khan, Fatima Ahsan, Shanawer Niaz, M. S. Al-Buriahi, Nora Mobark Farhan, Rawiyah Alkahtani, Sohail Mumtaz

**Affiliations:** a Institute of Physics, Khwaja Fareed University of Engineering and Information Technology Rahim Yar Khan 64200 Pakistan greatkhan17@gmail.com; b Department of Physics, Sakarya University Sakarya Turkey; c Department of Chemistry, College of Science, Princess Nourah bint Abdulrahman University P.O. Box 84428 Riyadh 11671 Saudi Arabia; d Department of Chemical and Biological Engineering, Gachon University 1342 Seongnamdaero, Sujeong-gu Seongnam-si 13120 Republic of Korea sohail.ahmed2015@gmail.com

## Abstract

Spintronics represents an area of study that makes use of the spin characteristic of the electron to control the behavior of quantum electronics devices relative to the typical charge electronics. In the present study, we carried out a detailed density functional theory-based analysis on the thermodynamic and mechanical stability of half-metallic ferromagnetic NiSc_2_X_4_ (X = S and Se) spinels under the influence of pressure. The formation energies and energy minimization in both the ferromagnetic and antiferromagnetic arrangements confirm the energetic stability in the ferromagnetic state. Also, there are no imaginary frequencies in the phonon dispersion and *ab initio* molecular dynamics simulations, showing that these two compounds are dynamically stable. It is clear that the mechanical behavior of these two materials is quite stable since they exhibit high Poisson's ratios and high bulk-to-shear modulus ratios. The Poisson's ratios of NiSc_2_S_4_ and NiSc_2_Se_4_ are found to be 0.309 and 0.295, respectively. The B_0_/G ratios of NiSc_2_S_4_ and NiSc_2_Se_4_ are found to be 2.287 and 2.114, respectively. Analysis of the electronic properties indicates that an increase in the applied pressure from 0 GPa to 4 GPa leads to a significant reduction in the direct band gap. Most importantly, NiSc_2_S_4_ becomes a half-metallic ferromagnet under increased pressure. The magnetic calculations indicate a magnetic moment of 2.00 *µ*_B_ per formula unit for both NiSc_2_S_4_ and NiSc_2_Se_4_, which originates mainly from the Ni atom. Also, an analysis of the thermodynamic properties of these two compounds, using the quasi-harmonic Debye approximation as implemented in the GIBBS2 program, shows lattice stiffening, phonon softening, and anharmonicity effects. The calculated entropy and Debye temperatures further confirm the stability and vibrational integrity of the system. Overall, these results show the potential of NiSc_2_X_4_ spinels in spintronic and magneto-electronic devices.

## Abstract 2

We examined the pressure-induced thermodynamic and mechanical characteristics of the half metallic spinels NiSc_2_X_4_ (X = S, Se). The stability of both materials has been checked from the perspective of energy and they show no imaginary phonon branches.

## Graphical abstract

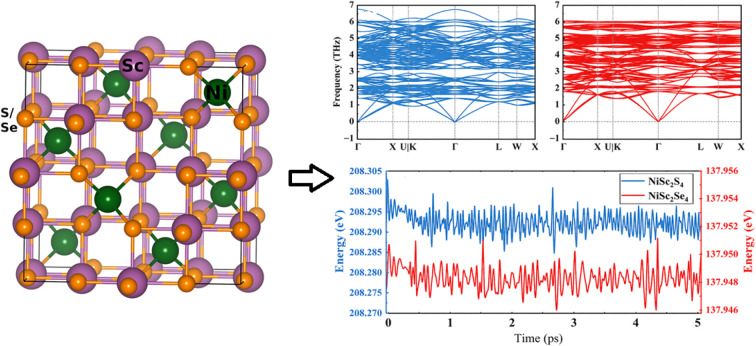

## 1 Introduction

Spintronics, which utilizes not only electron charge but also electron spin degrees of freedom, has become one of the most promising technologies for creating multifunctional devices with lower power requirements.^1^ Spin-based devices offer nonvolatility, ultrafast computation, and CMOS compatibility to resolve several problems encountered by conventional semiconductors.^2^ Significant results have been achieved in the area of magnetic tunnel junctions, spin–orbit torque switching and spin-transfer torque.^3^ Among all the known types of materials, spinel chalcogenides are considered particularly appealing because they can show the unique property of half-metallic ferromagnetism, distinguishing them from conventional materials such as metal oxides, Heuslers, and perovskites. The superior performance of these materials is related to their structural stability, their adjustable electronic characteristics, and the significant effects of electron correlation caused by transition-metal ions and, in some cases, rare-earth ions.^4,5^ Due to the flexibility of the spinel structure, which contains multiple cation positions in crystals, the magnetic and electronic properties of spinel chalcogenides can be easily tuned, while other types of families, such as perovskites and Heusler compounds, face problems of phase instabilities.^6^ In addition, spinel chalcogenides possess strong p–d and p–f orbital hybridizations, allowing for the stabilization of half-metallicity and complete spin polarization necessary for spintronic applications, together with low thermal conductivity, providing better thermoelectric behavior. Thus, spinel chalcogenides can be regarded as a promising material type for future spintronic and transport devices.^7,8^

From the variety of material groups examined for possible use in spintronic devices, special interest is paid to half-metallic compounds, which possess unique electronic structures in which one spin channel is metallic and the second is a semiconductor/insulator. As a result, half-metallic materials show full spin polarization at the Fermi level, which is important for effective spin transport and detection. Examples of half-metallic ferromagnets include half-metallic Heusler alloys, spinel chalcogenides, manganates and some transition-metal oxides.^9^ Spinels represent a versatile class of materials that are central to various modern technologies ranging from green energy storage to microelectronics.^10^ Their remarkable structural diversity in terms of compositions and site occupancy enables them to host large magnetic and non-magnetic cations, unlocking complex exchange-coupling pathways. These materials are widely explored for their distinct physical properties, which include mechanical robustness, magnetoelectric or multiferroic behavior suitable for different applications.^11,12^ While traditional transition-metal spinels have been thoroughly studied, the exploration of scandium-based chalcogenide spinels remains unexplored. The incorporation of both magnetic and non-magnetic species in spinels allows the exchange interactions to be confined to a particular site, which drastically minimizes the long-range magnetic interactions, thereby allowing the regulation of the crystal environment and enabling quantum phenomena.^13,14^ In this context, NiSc_2_X_4_ systems could provide an optimal platform to overcome the electronic limitations of conventional wide-bandgap spinels. Moreover, the high pressure sensitivity of their structure and strong covalent bonding provide additional opportunity for tuning the electronic and magnetic properties by applying pressure.^15^

In addition, Özdemir and Balmumcu (2024) carried out a computational study on the electronic and mechanical properties of VCo_2_O_4_ with the use of an approach similar to that employed by Ak *et al.*^16^ They confirmed the presence of half-metallic ferromagnetism with a magnetic moment of 10.0 *µ*_B_ per formula unit, an equilibrium lattice constant of 8.34 Å, and a widening of the band gap from 1.262 electron volt (GGA) to 1.286 electron volt (GGA + *U* = 4 eV). Also, the material in question was mechanically stable and ductile, with a Debye temperature of 565 K.^17,18^ Inspired by these studies, Özdemir *et al.* (2024) investigated another spinel, VRu_2_Br_4_, with a similar approach. This material has a ferromagnetic ground state with a lattice constant value close to 10.93 Å and a total magnetic moment of 10.0 *µ*_B_ per formula unit. The band gap of VRu_2_Br_4_ is easily tuned and varies from 0.210 eV (GGA) to 1.718 eV (GGA + *U* = 3 eV). It can be stated that the material under consideration is ductile at zero pressure (B/G = 2.48, *ν* = 0.322), but an increase in applied pressure up to 10 GPa leads to brittle behavior along with a decrease in the Debye temperature to 257.22 K.^19,20^ A study carried out by Raza *et al.*^21^ revealed direct band gaps in MnSc_2_X_4_ (X = S and Se) spinels, static dielectric constants of 6.5 and 8.5, and Seebeck coefficient values in the range 242–251 µV K^−1^. Furthermore, these materials show ferromagnetic ordering with relatively large local magnetic moments as well as thermodynamic and mechanical stability, thus being interesting candidates for optoelectronics and thermoelectronics.^22–24^ Jamaï *et al.* (2024) studied the band structure of XIn_2_M_4_ (X = Cd and Zn; M = S, Se and Te) spinels with indirect band gaps varying from 2.294 eV (CdIn_2_S_4_) to 0.219 eV (ZnIn_2_Te_4_). These materials demonstrate high absorption in the UV-visible region and good structural stability. In addition, the values of the thermoelectric figure of merit in the range of 0.725–0.803 at 300 K prove their possible usage in thermoelectric devices.^25^

The present work provides a thorough investigation of pressure-induced electronic transitions and mechanical anisotropy profiles of stable NiSc_2_X_4_ (X = S and Se) spinels from the first-principles perspective. It is noted that structural stability, elastic anisotropy, pressure-induced band gap tuning, magnetism, and thermodynamic aspects were studied in the context of the quasi-harmonic Debye approximation model to investigate the prospective application of NiSc_2_X_4_ compounds in magneto-electronics. Combining all of these features, the work investigates the prospects of the materials for magneto-electronics, providing some information about their multistate pressure-driven properties that have not been studied enough in the modern literature. Moreover, strong hybridization of the Ni 3d states and the chalcogen p (S/Se) states is responsible for the electronic band structure formation and carrier scattering reduction, which makes possible the appearance of half-metallic properties and close to 100% spin polarization. Therefore, the Ni-containing spinel chalcogenides can be regarded as an interesting material class for prospective spintronic applications.

## 2 Computational methodology

In the current work, spin-polarized density functional theory (DFT) calculations have been carried out to investigate the structural, electronic, magnetic, and thermodynamic properties of spinel-type chalcogenides NiSc_2_X_4_ (X = S and Se). All the calculations were performed using the FP-LAPW + lo method, as implemented in the WIEN2k code.^26,27^ This method is suitable for accurately describing transition metals because of the all-electron treatment of muffin-tin and interstitial space. To carry out structural optimization and evaluate the mechanical properties of the compounds, the PBEsol exchange–correlation functional within the GGA approximation was used. The PBEsol exchange–correlation functional has proved to be quite reliable for the prediction of equilibrium lattice parameters and cohesive properties of crystalline solids. The band gap problem inherent in GGA functional approximations was tackled by using the TB-mBJ exchange potential to evaluate the electronic structure of the compounds under investigation.^28^ The application of the PBEsol and TB-mBJ functionals provides a realistic description of the ground-state structural properties and electronic behavior of NiSc_2_X_4_ spinels.

Convergence was reached by choosing proper computational parameters. The value of *R*_MT_*K*_max_ is 8, according to the accepted criterion of convergence for systems that include transition metals and heavier elements. Muffin-tin radii were set up as *R*_MT_ (Ni) = 2.30, *R*_MT_ (Sc) = 1.80, *R*_MT_ (S) = 1.85, and *R*_MT_ (Se) = 2.10 to prevent overlapping of adjacent spheres and to provide the necessary flexibility of the basis sets. The energy cut-off value was chosen to be −9.0 Ry to distinguish core and valence states. Thus, proper descriptions of the semi-core states were obtained. The value of the maximal quantum number of angular momentum for the partial wave expansion inside the muffin-tin spheres was set as *l*_max_ = 10 for adequate treatment of the contribution of d- and f-electrons. For the Fourier expansion of the charge density, *G*_max_ was set as 22 Ry. For Brillouin zone sampling, the Monkhorst–Pack grid was used, with 1000 *k*-points in the grid, providing convergent total energies. Further, increasing the *k*-point density does not produce any change in the total energy, demonstrating convergence regarding reciprocal space sampling. The thermodynamic properties of NiSc_2_X_4_ were calculated using the quasi-harmonic Debye model, which is realized in the Gibbs2 code of the WIEN2k package.^29^

## 3 Results and discussion

### 3.1 Structural analysis

**Fig. 1:**
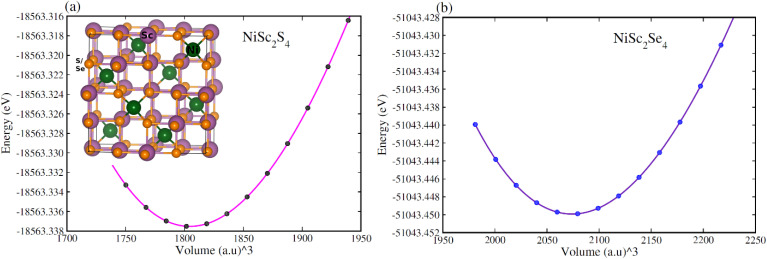
Unit cell (inset) and volume optimization plots for the (a) NiSc_2_S_4_ and (b) NiSc_2_Se_4_ spinels in FM spin orientations.

The structure of the cubic NiSc_2_X_4_ (X = S and Se) spinels is illustrated in Fig. 1(a) with a ball-and-stick model and a polyhedral representation. In this structure, Ni atoms (green) are located at the A tetrahedral sites, whereas Sc atoms (pink) are at the B octahedral sites, forming a face-centered cubic lattice, coordinated with anions (yellow) S or Se to create the structure. The crystals crystallize in the *Fd*3̄*m* (No. 227) space group.^30^ Spin-polarized density functional theory (DFT) total-energy calculations were used to obtain the magnetic ground state, considering ferromagnetic (FM) and antiferromagnetic (AFM) states. Total energy calculations were carried out with respect to the volume of the unit cell, and the energy–volume (*E*–*V*) curves for NiSc_2_S_4_ and NiSc_2_Se_4_ compounds in the FM state are presented in Fig. 1(a) and (b). The FM state is more thermodynamically stable because of its low total energy compared to that of the AFM state (see Table 1). The energy difference (Δ*E* = *E*_AFM_ − *E*_FM_) testifies to the thermodynamic stability of the FM state. The thermodynamic stability of these spinels was estimated by calculating the formation enthalpy (Δ*H*_f_) according to: ^31^

1 Δ*H*_f_ = *E*_Total_ (Ni_*l*_Sc_*m*_Z_*n*_) − *lE*_Ni_ − *mE*_Sc_ − *nE*_X_

where *E*_Total_ is the ground-state total energy of the spinel per formula unit and *E*_Ni_, *E*_Sc_, and *E*_X_ are the ground-state energies of pure elements Ni, Sc, and S/Se, respectively. In this regard, *l*, *m*, and *n* are integers representing the number of Ni, Sc, and S/Se, respectively, in the spinel formula unit. Table 1 provides the formation energies of the spinels, with both being negative relative to their respective elemental counterparts. Moreover, Δ*H*_f_ is slightly less negative for NiSc_2_Se_4_ as compared to NiSc_2_S_4_, meaning that a slight instability in comparison to the former arises from the substitution of S for Se atoms, which results in longer distances between ions due to the higher ionic radii of Se^2−^ anions in comparison to those of S^2−^. The equilibrium lattice parameter *a*_0_ and the equilibrium value of bulk modulus *B*_0_ were calculated during volume optimization within the framework of the FM state. For the description of the volume-energy dependence, we used the Murnaghan equation of state in combination with the PBEsol-GGA functional to obtain the equilibrium volume. It is likely that the lattice constant (*a*_0_) of the two spinels is increased when going from NiSc_2_S_4_ to NiSc_2_Se_4_ because of the substitution of the smaller ions by larger ones. In agreement with this, the same trend was predicted for similar spinels, namely, CoSc_2_S_4_ and CoSc_2_Se_4_, using first-principles calculations.^32^ Consequently, the bulk modulus *B*_0_ gets lower due to the increased compressibility of NiSc_2_Se_4_. The negative correlation of the lattice parameter and the bulk modulus can be explained by the solid-state theory in such a way that larger unit cell volumes imply weaker interatomic interactions and softer mechanical properties of materials.

**Table 1:** Ground-state lattice constants *a*_0_ (Å), bulk modulus *B*_0_ (GPa), energy difference (Δ*E* = *E*_AFM_ − *E*_FM_) and enthalpy of formation Δ*H*_f_ (eV) calculated for the NiSc_2_S_4_ and NiSc_2_Se_4_ spinels

Composition	*a*_*0*_ (Å)	*B*_0_ (GPa)	Δ*E* (meV)	Δ*H*_f_ (eV)
NiSc_2_S_4_	10.23	71.16	38.64	−1.06
NiSc_2_Se_4_	11.71	64.19	31.58	−0.82

**Fig. 2:**
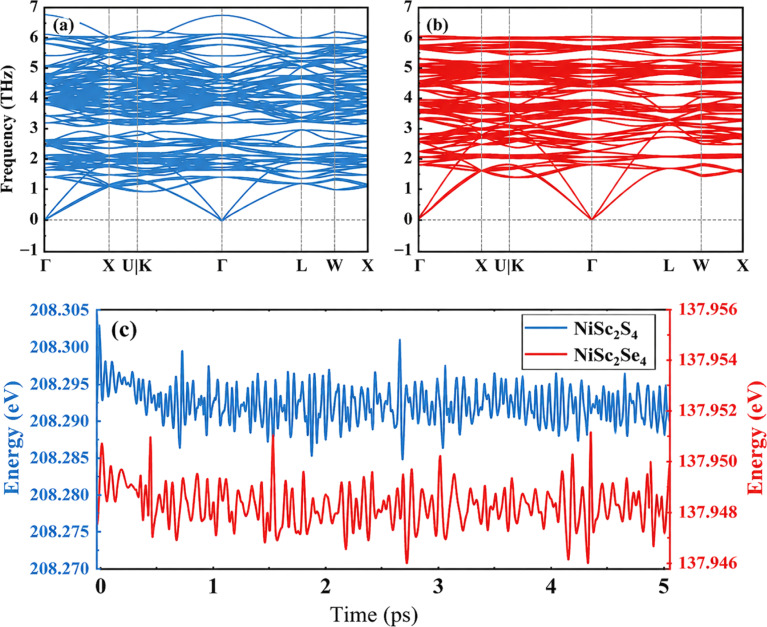
Phonon dispersion plot of the (a) NiSc_2_S_4_ and (b) NiSc_2_Se_4_ spinels, and (c) AIMD plot for both spinels.

The phonon properties were calculated using the Phonopy code.^33^ Dynamical stability is defined through the requirement for an absence of phonon instability at the *Γ*-point in the case of cubic systems; analyses performed in the Brillouin zone of the unit cell revealed the fulfillment of this condition for the spinel structures under study. Therefore, phonon dispersion spectra shown in Fig. 2(a and b) demonstrate the dynamic stability of the optimized structures. There are seven atoms in the primitive cell of each spinel and, hence, there are three acoustic and eighteen optical phonon branches, as seen in Fig. 2(a and b). The absence of gaps between the optical branches proves that the mass distribution of the atoms is regular. Additional phonon calculations made on the basis of pre-optimized structures provide additional proof that the dynamic stability of these compounds is suitable for practical application in spintronics. In order to estimate the thermal stability of the materials, AIMD simulations were conducted using the Langevin (NPT) thermostat at 300 K.^34^ A time step of 1 fs and a total simulation time of 5 ps were used, and the energy-time plots are shown in Fig. 2(c). Total energy oscillates about a constant value, indicating no evidence of structural collapse and chemical dissociation; therefore, the thermal stability of the material is proven. Oscillations of the total energy at 300 K mean that the structure of NiSc_2_X_4_ remains stable at ambient temperature, while being more stable at lower temperatures.

### 3.2 Mechanical properties

To reveal the mechanical properties of the NiSc_2_S_4_ and NiSc_2_Se_4_ spinels, the second-order elastic constants C_11_, C_12_, and C_44_ were calculated under different pressures. These elastic constants define the elastic characteristics of cubic crystals, allowing the principal mechanical moduli to be derived.^35^ The calculated elastic constants satisfy the Born stability criteria for cubic crystals, namely C_11_ > 0, C_11_ − C_12_ > 0, and C_11_ + 2C_12_ > 0. Thus, both materials are mechanically stable within the analyzed pressure range. Based on the second-order elastic constants, the bulk modulus *B*, shear modulus *G*, and Young's modulus *E* were calculated by using the Voigt–Reuss–Hill averaging scheme, with the obtained values of 0, 2, and 4 GPa given in Table 2. It can be seen that the bulk modulus derived from the elastic constants at 0 GPa is larger than the value obtained from the equation of state (EOS). This difference is a known artifact of the difference in the sampling methods used in the two schemes, where the bulk modulus obtained from the elastic coefficients represents localized, infinitesimal directional strains at the equilibrium geometry compared to global hydrostatic compression used in the equation of state formula. In this regard, it is possible to claim the mechanical stability of the analyzed structures and their resistance to volume deformation. To identify whether the analyzed materials are ductile or brittle, the B/G ratio and Poisson's ratio *ν* were calculated. According to the traditional criteria, if B/G is higher than 1.75 and Poisson's ratio is larger than 0.26, the material is ductile. In this case, at 0 GPa, the B/G ratios are 2.287 and 2.114, while Poisson's ratios are 0.309 and 0.294 for NiSc_2_S_4_ and NiSc_2_Se_4_, respectively. Thus, it can be concluded that both materials possess ductile mechanical properties, which is beneficial for their use in thermomechanical energy conversion devices and robust electronics. The high values of B/G and *ν* at all considered pressures confirm their ductility and mechanical strength. As for NiSc_2_X_4_ spinels, similar to other ductile, mechanically and thermodynamically stable compounds, recently discovered MAX phases and their solid solutions possess mechanical robustness and ductility, where there is a strong dependence between elastic constants and ductility parameters.^36,37^ Therefore, it is possible to draw conclusions about the ductility and mechanical robustness of NiSc_2_X_4_ spinels, which can be considered similar to other ductile, mechanically and thermodynamically stable compounds.

**Table 2:** Calculated elastic (C_11_, C_12_, C_44_) parameters, bulk modulus (*B*), shear modulus (*G*), Young's modulus (*Y*), Poisson ratio (*ν*) and Pugh's (B/G) ratio for the NiSc_2_S_4_ and NiSc_2_Se_4_ spinels at different pressures (0 GPa, 2 GPa and 4 GPa)

Pressure (GPa)		C_11_ (GPa)	C_12_ (GPa)	C_44_ (GPa)	*B* (GPa)	*G* (GPa)	*Y* (GPa)	*ν*	B/G
NiSc_2_S_4_	0	173.37	53.36	31.41	93.37	40.83	106.89	0.309	2.287
	2	223.05	62.81	39.06	116.23	52.31	136.45	0.304	2.222
	4	292.58	76.86	49.47	148.77	67.98	176.99	0.301	2.188
NiSc_2_Se_4_	0	160.52	32.39	23.39	75.10	35.50	92.01	0.295	2.114
	2	193.63	55.96	31.11	101.85	43.02	113.14	0.314	2.367
	4	212.22	76.74	38.09	121.90	48.06	127.44	0.326	2.536

**Fig. 3:**
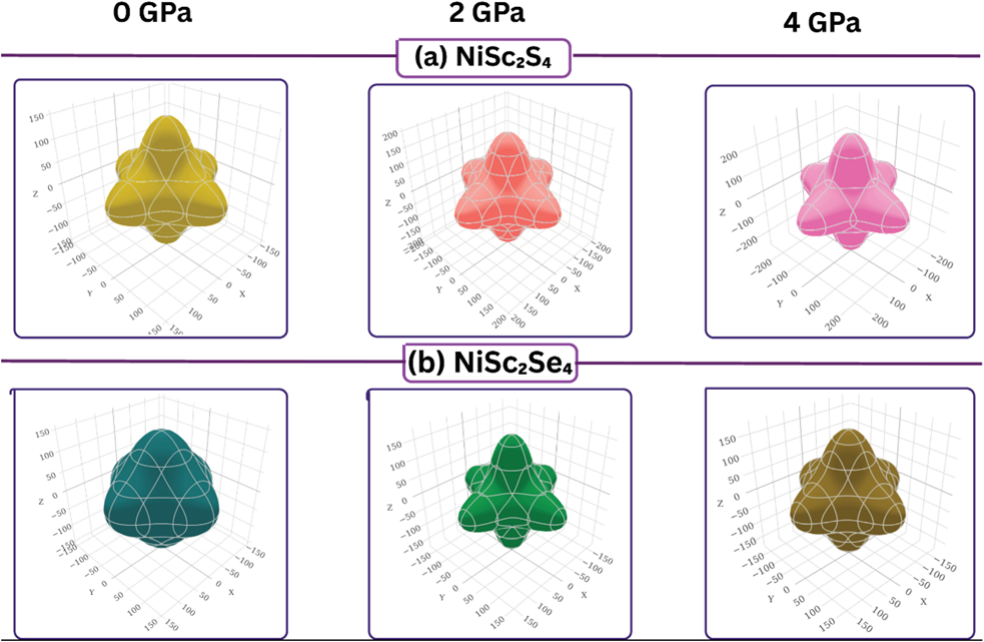
3D plots of the Young modulus for the (a) NiSc_2_S_4_ and (b) NiSc_2_Se_4_ spinels at 0, 2 and 4 GPa.

**Fig. 4:**
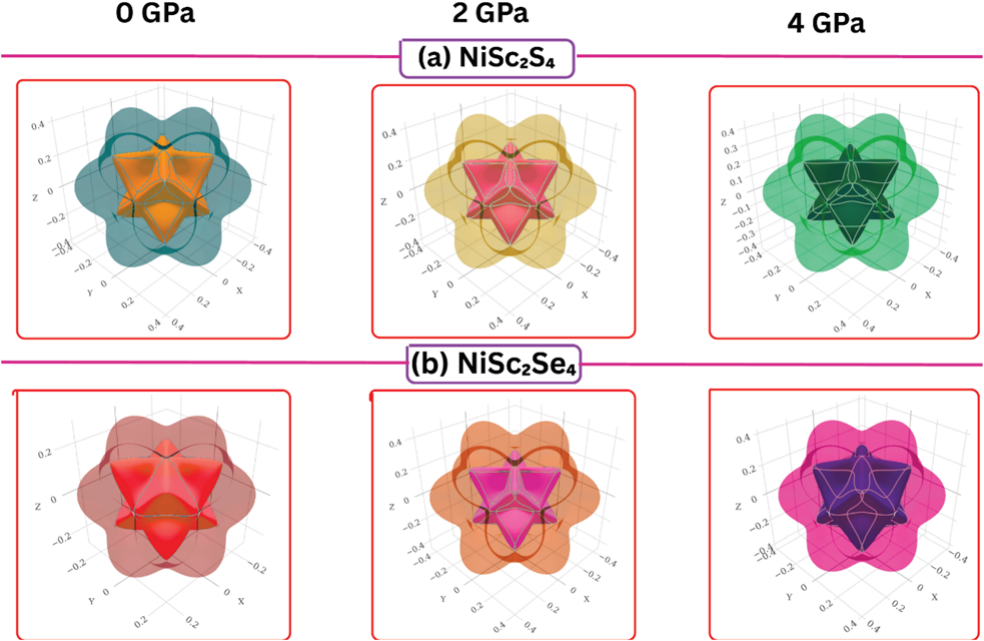
3D plots of Poisson's ratios for the (a) NiSc_2_S_4_ and (b) NiSc_2_Se_4_ spinels at different pressures (0 GPa, 2 GPa and 4 GPa).

Elastic anisotropy is one of the basic features of crystalline solids. It stems from the orientation-dependent character of their mechanical properties, because elastic moduli depend on the orientation of the crystal.^38^ For isotropic media, by contrast, all mechanical characteristics are the same for any direction. Elastic properties do not depend on the direction chosen. The assessment of the elastic anisotropy of the material was carried out by constructing three-dimensional graphs of Young's modulus (*Y*) and Poisson's ratio (*ν*). These surfaces for NiSc_2_X_4_ are presented in Fig. 3 and 4. The 3D Young's modulus surfaces of NiSc_2_S_4_ and NiSc_2_Se_4_ at 0, 2 and 4 GPa are presented in Fig. 3. The surface area of the Young's modulus increases with increasing pressure for NiSc_2_S_4_. This means that the stiffness of the compound grows, and the resistance to elastic deformation increases. The growth of the radial extent at 2 and 4 GPa is the consequence of strengthening of atomic bonds and a larger Young's modulus value. Despite some anisotropy, the increased size of the surface demonstrates the growing mechanical rigidity due to the compression. In turn, NiSc_2_Se_4_ has a smaller surface area and Young's modulus compared with NiSc_2_S_4_, and it is more sensitive to pressure. Under ambient conditions, the surface of the material is nearly isotropic, but the growth of pressure leads to the appearance of anisotropic features.

3D visualization of Poisson's ratios for NiSc_2_S_4_ and NiSc_2_Se_4_ under 0, 2, and 4 GPa (Fig. 4) demonstrates pressure-dependent elastic anisotropy. The morphology of NiSc_2_S_4_ Poisson's ratio distributions is very complex and non-spherical, suggesting a strong directionality of the material's transverse strain. An increase in pressure from 0 to 4 GPa leads to a moderate expansion of the surface, which maintains its symmetry, thus pointing to an increase in the value of Poisson's ratio and elastic coupling between longitudinal and transverse strains. Poisson's ratio distributions for NiSc_2_Se_4_ show anisotropic features similar to those of NiSc_2_S_4_; however, the degree of the anisotropy is slightly lower for the selenium-based compound. The gradual increase in surface size with increasing pressure suggests an increase in Poisson's ratio upon compression and increased probability of lateral deformation upon axial loads. Elastic anisotropy of the sulfide NiSc_2_S_4_ can be explained by the more directional nature of bonds formed by Ni and Sc atoms with S, which lead to significant differences in stiffness and deformation resistance among different crystallographic directions.^39^ The introduction of selenium makes the NiSc_2_Se_4_ lattice softer and less directional, thus contributing to an anisotropy reduction. This trend from NiSc_2_S_4_ to NiSc_2_Se_4_ is an example of a direct structure–property relationship, where the influence of chalcogen substitution manifests itself not only on equilibrium lattice parameters and bonding structure but also on elastic anisotropy.^40^ Understanding such anisotropic behavior is crucial for assessing mechanical stability and practical applications of such compounds in technological systems, where elastic anisotropy plays an important role (magnetostriction, spintronics, and pressure-tunable optoelectronics, *etc.*).

### 3.3 Electronic structure analysis of NiSc_2_X_4_

**Fig. 5:**
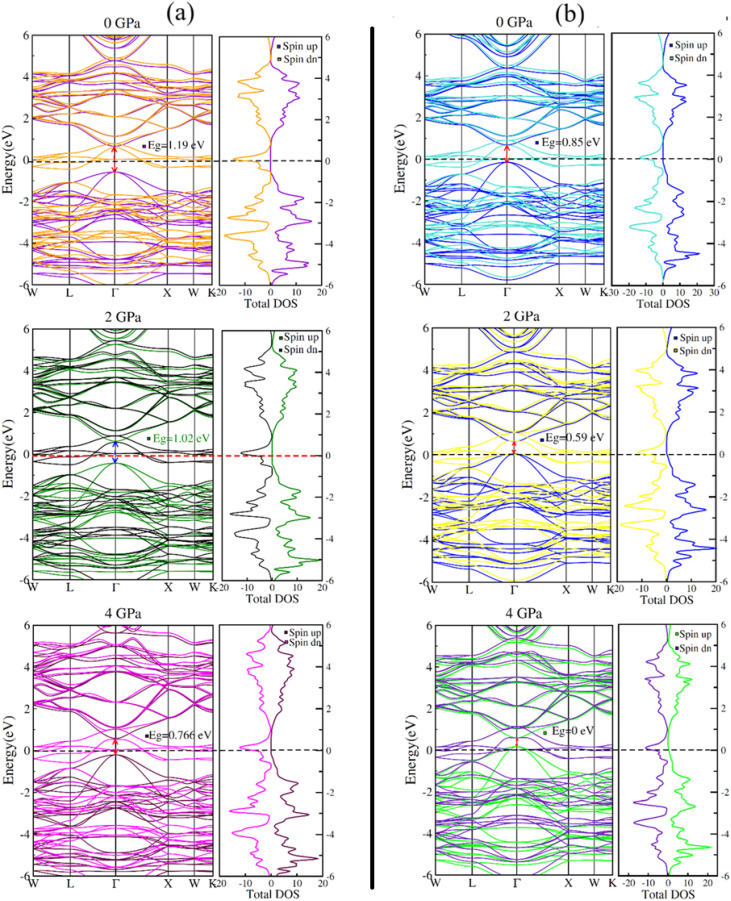
Electronic band structures and total DOS for the (a) NiSc_2_S_4_ and (b) NiSc_2_Se_4_ spinels at different pressures (0 GPa, 2 GPa and 4 GPa).

The electron configuration of a particular material determines its intrinsic properties, which then define its applications in the field of electronics. Among many others, the energy band gap is considered an important factor for such applications as memory elements, smart electronics, and spintronics. Controlling the energy band gap enables control of the transportation of charges and spins inside the system, providing functionality such as information storage, signal processing, and spin manipulation. In order to describe the electronic properties of NiSc_2_X_4_ (X = S and Se) correctly, the TB-mBJ potential is used, as presented in Fig. 5(a) and (b). In Fig. 5(a), the electronic configuration is studied at 0 GPa, where it is found that the band edges of NiSc_2_S_4_ are aligned at the *Γ* point, meaning the material behaves as a direct band gap semiconductor in the spin-down channel, whereas the spin-up channel is metallic. It should be noted that the dispersion of the band structure is rather moderate, especially for the conduction part.^41^ The spin-resolved band shows asymmetry between the spin-up (orange) and spin-down (purple) states near the Fermi energy level (*E*_F_) due to the magnetic ordering. The spin splitting occurs as a result of exchange interaction in the Ni 3d orbitals, characteristic of the magnetic spinel materials. The application of a pressure of 2 GPa causes significant changes in the electronic structure of NiSc_2_S_4_, as shown in Fig. 5(a). The direct band gap becomes smaller because of the changes in the positions of the valence band maximum (VBM) and conduction band minimum (CBM), lying at the *Γ* point; in addition, the CBM in the spin-down channel moves downwards while the VBM in the spin-up channel increases slightly.^42^ This means an increase in the orbital hybridization and electron delocalization caused by the lattice compression. The enhanced orbital overlap of Ni–S–Sc increases the coupling of the Ni-3d, Sc-3d, and S-3p states, resulting in broadening of the conduction band. The electronic structure modification is also observed at 4 GPa, as can be seen in Fig. 5(a). The direct band gap becomes smaller, while the *Γ*-point CBM approaches *E*_F_ and the *Γ*-point VBM stays the same, retaining the direct band character. Notably, the spin polarization is strong over the whole Brillouin zone, which suggests that the magnetic exchange interaction is preserved in the presence of the pressure, most probably *via* Ni–S–Ni superexchange pathways inherent to the spinel structure.

Fig. 5(b) shows the spin-polarized band structure of NiSc_2_Se_4_ under 0, 2, and 4 GPa pressures based on the TB-mBJ potential. Energy dispersion was determined along the high-symmetry lines of the Brillouin zone of the FCC crystal structure, providing valuable information about the changes in magnetic and electronic properties as a result of the pressure application. It can be seen that NiSc_2_Se_4_ acts as a direct semiconductor at 0 GPa, where both VBM and CBM occur at the *Γ* point. With the application of 2 GPa of pressure, the width of the band gap reduces due to mixing between the Ni 3d, Sc 3d, and Se 4p orbitals. As seen in the spin-down band structure, the CBM moves down while the VBM moves up, which indicates the higher conductivity of this material.^43^ At 4 GPa (Fig. 5(c)), the band gap becomes even smaller while the material exhibits metallic behavior. The systematic narrowing of the direct band gap, as well as spin asymmetry and half-metallic properties that appear due to it, suggest the possibility of using NiSc_2_Se_4_ as a quantum material for spintronic and energy purposes.

**Fig. 6:**
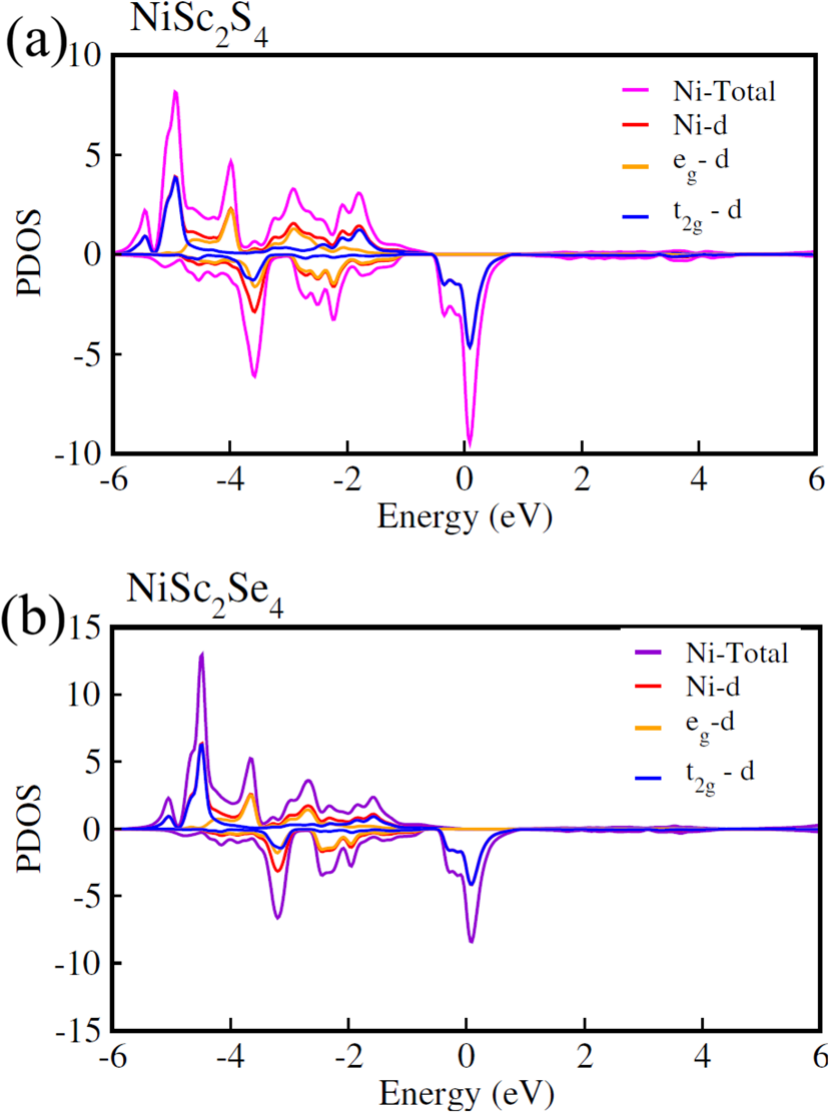
Partial DOS of d orbitals for the (a) NiSc_2_S_4_ and (b) NiSc_2_Se_4_ spinels in spin-up (↑) and spin-down (↓), computed with the mBJ potential at 0 GPa.

In order to understand the pressure-induced electronic properties of the NiSc_2_S_4_ and NiSc_2_Se_4_ spinel systems, the total and partial density-of-state (DOS) plots, as shown in Fig. 5(a, b), 6(a and b), were examined. The total DOS of both materials at 0, 2, and 4 GPa are plotted, including the spin-up and -down contributions separately. For NiSc_2_S_4_, the significant difference between the spin-up and spin-down states at 0 GPa is a result of spin polarization of the material at the Fermi energy (*E*_F_). The *E*_F_ lies inside the band gap for the spin-up case, while the spin-down case remains semiconducting. The increase in pressure results in the shift of the band edges and the decrease of the band gap width for both spin cases.^44^ This band gap narrowing, combined with the increased DOS at the *E*_F_ point, suggests increased electron delocalization and pressure-induced changes in the magnetic exchange interaction through a Ni–S–Ni super-exchange mechanism.

On the other hand, in NiSc_2_Se_4_, both the exchange splitting and the spin polarization are lower, as seen in Fig. 5(b). For a pressure of 4 GPa, the density of states (DOS) exhibits a nonzero value of states at the Fermi level for both spins, which shows the semi-metallic/weakly metallic nature of the material. With increasing pressure, there will be only minor changes in the DOS structure, which suggests that NiSc_2_Se_4_ retains its low spin state. This can be due to reduced hybridization of Ni 3d with Se 4p, in addition to band broadening caused by the larger atomic size and the lower electronegativity of Se.

The partial density of states (PDOS) is depicted in Fig. 6(a and b) for the system at 0 GPa pressure, whereby the DOS is partitioned into the orbital character of the Ni and chalcogen elements. In the case of NiSc_2_S_4_, near *E*_F_, the density of states is made up of Ni 3d states that are split into two degeneracy levels due to the octahedral crystal field of ligands. Exchange splitting can be seen very clearly as the spin-up states in the e_g_ manifold are deep below *E*_F_, and the t_2g_ states are lifted above *E*_F_, in agreement with the high-spin state of Ni^2+^. There is strong hybridization between the S 3p states and Ni 3d states in the valence region (−6 to 0 eV), thus indicating considerable p–d covalency for both the magnetism and conduction processes. In the case of NiSc_2_Se_4_, the Ni 3d states have higher delocalization, with weak exchange splitting. The Se 4p states occupy a larger energy range compared to the S 3p, and there is no clear hybridization between the Se 4p and Ni 3d states.^45^

The overall trend from the electronic structure calculations suggests that the NiSc_2_S_4_ material is highly spin-polarized, with a half-metallic property that is pressure-sensitive, while NiSc_2_Se_4_ possesses weaker magnetic properties that are localized. The application of pressure results in an alteration of the electronic environment in both materials, but there is a larger response in the sulfide due to compression of the lattice that leads to orbital hybridization and magnetic coupling. This disparity provides proof of the importance of the chalcogenide ion in determining the electron localization/delocalization and stability of the magnetic order in spinel structures. This information is fundamental in designing new materials with desirable magnetic and electrical characteristics.

### 3.4 Magnetic properties

The magnetic characteristics of spinel-type materials continue to be one of the key aspects to consider when evaluating their potential applicability to the development of spintronic and magneto-electronic technologies. In the current work, the magnetic characteristics of NiSc_2_X_4_ (X = S and Se) were studied based on the analysis of both total and site-specific magnetic moments depending on applied pressure; the results are given in Table 3 in units of Bohr magnetons (*µ*_B_). For both of the studied materials, NiSc_2_S_4_ and NiSc_2_Se_4_, the total magnetic moment of the unit cell is about 2.000 *µ*_B_ and results from partially filled 3d orbitals of Ni ions located in tetrahedral sites.^46^ The total magnetic moment of NiSc_2_S_4_ does not change significantly at 0–4 GPa and stays close to 2 *µ*_B_, indicating high stability of the half-metallic ferromagnetism in this compound under applied pressure. It has been found that the local moment of Ni ions decreases from 1.4657 *µ*_B_ at 0 GPa to 1.3554 *µ*_B_ at 4 GPa, whereas the magnetic moments of Sc and S ions increase slightly with increasing pressure. Thus, the observed decrease in the local moment is related to an increase in orbital hybridization and exchange interaction between the Ni 3d states and the surrounding atoms due to a decrease in the lattice parameters.^47^ For NiSc_2_Se_4_, the total magnetic moment decreases slowly from 2.00069 *µ*_B_ at atmospheric pressure to 1.91202 *µ*_B_ at 4 GPa. At the same time, the local moment of Ni ions decreases from 1.3905 *µ*_B_ to 1.2769 *µ*_B_, whereas the magnetic moments of Sc and Se ions undergo minor changes. The non-integer value of the total magnetic moment at elevated pressures can be associated with the decreasing half-metallic nature of the material due to pressure-induced changes in the electronic band structure and increasing p–d hybridization.^48^

**Table 3:** Total and local magnetic moments (in Bohr magnetons) calculated for the NiSc_2_S_4_ and NiSc_2_Se_4_ spinels at different pressures (0 GPa, 2 GPa and 4 GPa)

Pressure (GPa)		*M*_Total_	*M*_Ni_	*M*_Sc_	*M*_X_
NiSc_2_S_4_	0	2.00	1.47	0.01	0.078
	2	2.00	1.42	0.02	0.081
	4	2.00	1.35	0.02	0.084
NiSc_2_Se_4_	0	2.00	1.39	0.03	0.083
	2	1.98	1.27	0.05	0.086
	4	1.91	1.28	0.04	0.083

### 3.5 Thermodynamic properties

**Fig. 7:**
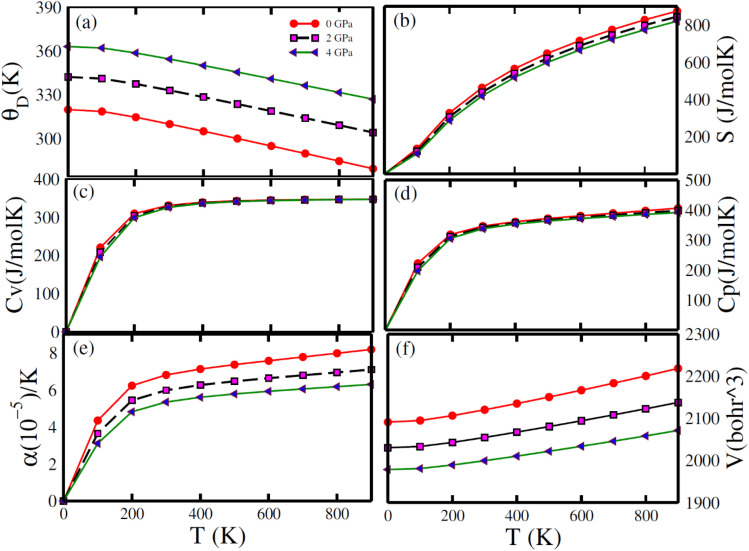
Calculated values of (a) Debye temperature (*θ*_D_), (b) entropy (*S*), (c) specific heat (*C*_v_), (d) *C*_p_, (e) thermal absorption and (f) volume (*V*) for the NiSc_2_S_4_ spinel at 0 GPa, 2 GPa and 4 GPa.

**Fig. 8:**
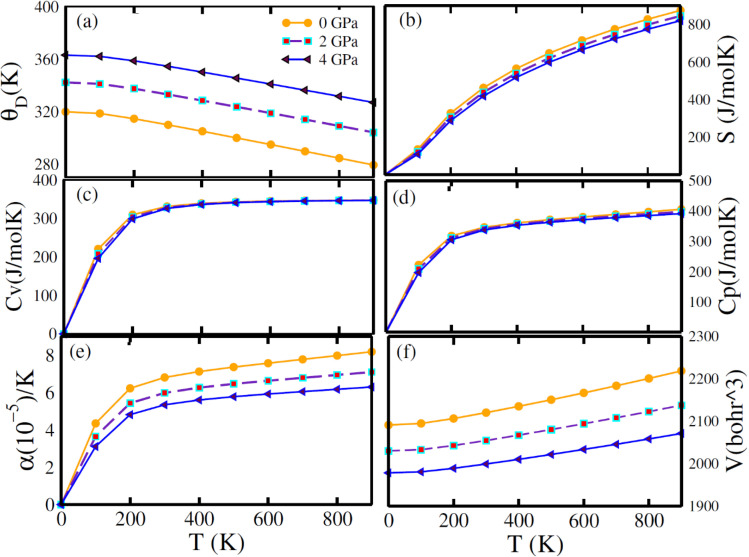
Calculated values of (a) Debye temperature (*θ*_D_), (b) entropy (*S*), (c) specific heat (*C*_v_), (d) *C*_p_, (e) thermal absorption and (f) volume (*V*) for the NiSc_2_Se_4_ spinel at 0 GPa, 2 GPa and 4 GPa.

In order to analyze the temperature and pressure dependence of various thermodynamic properties of the NiSc_2_X_4_ (X = S and Se) spinel structures, the quasi-harmonic Debye model was used with the GIBBS2 code.^49^ This technique facilitates the accurate computation of important thermodynamic parameters, such as entropy (*S*), heat capacity (*C*_v_), Debye temperature (*θ*_D_), and the coefficient of thermal expansion (*α*) from 0 to 800 K, for hydrostatic pressures of 0, 2, and 4 GPa, as depicted in Fig. 7(a–f) and 8(a–f). The thermodynamic dependencies up to 600 K were found using the quasi-harmonic approximation; however, despite the fact that such a temperature scale allows one to find general tendencies, it is limited in terms of the actual physical values—the melting and Curie temperatures of NiSc_2_X_4_. Therefore, the values found at higher temperatures can be considered only as theoretical estimates. The obtained thermodynamic data provide useful information on anharmonic vibrations, mechanical stability, and phonon dynamics in spinels, which is important for evaluating their properties as materials for energy applications.^50^

Among the calculated thermodynamic properties, entropy (*S*) appears to be especially meaningful, as it describes the amount of disorder and the number of phonons in the lattice. Indeed, as can be seen from Fig. 7 and 8(b), *S* exhibits a monotonic dependence on *T* and approaches zero at the limit of 0 K, which corresponds to the formulation of the 3rd law of thermodynamics.^51^ The increase in entropy values at low *T* is explained by fast activation of low-energy phonon modes and saturates at high temperatures due to the filling of all vibrational states. Dependence of S on the pressure is easier to observe for NiSc_2_Se_4_, especially at high temperatures. For instance, at *T* = 300 K and *P* = 0 GPa, the entropy of NiSc_2_S_4_ is 430 J mol^−1^ K^−1^, whereas the entropy of NiSc_2_Se_4_ is 465 J mol^−1^ K^−1^, as shown in Fig. 7 and 8(a). Such an increase from S-based to Se-based compounds is associated with the higher atomic mass of Se and a greater number of vibrational degrees of freedom.^52^

The temperature and pressure dependence of entropy are inherently connected with the temperature dependence of the Debye temperature *θ*_D_, which represents the boundary between vibrational modes and lattice stiffness. As seen from Fig. 7 and 8(a), the Debye temperature *θ*_D_ remains practically constant down to cryogenic temperatures, but starting from around 320 K for NiSc_2_S_4_ and 316 K for NiSc_2_Se_4_, its linear decay can be noted due to the softening of phonons and increasing anharmonicity of the crystal lattice.^53^ Conversely, upon applying external pressure, the decrease in *θ*_D_ is almost linear for NiSc_2_S_4_, while an increase in *θ*_D_ is observed for NiSc_2_Se_4_, which means stronger interaction between atoms and increased stiffness of the crystal lattice. The increase in *θ*_D_ for NiSc_2_Se_4_ with pressure corresponds with decreasing entropy and points to a more ordered and rigid structure under high-pressure conditions. The Debye temperatures at 300 K and 0 GPa are 312 K for NiSc_2_S_4_ and 308 K for NiSc_2_Se_4_, as expected, because there is an inverse correlation between Debye temperature and atomic mass.

The correlation between *S* and *θ*_D_ determines the nature of the heat capacity *C*_v_, which depends principally on phonon excitations. The T^3^ dependence of *C*_v_ in the low-temperature regime, as predicted by the Debye theory, and the asymptotic behavior towards the classical Dulong–Petit limit in the high-temperature regime, when all phonon modes are thermally populated, can be seen from Fig. 7 and 8(c).^54^ Quantum-classical transition in *C*_v_ becomes especially pronounced at temperatures lower than about 220 K; above this temperature, it rises much more slowly and finally reaches a saturation point. The effect of pressure on *C*_v_ is rather weak and results in a decrease caused by a reduction in the number of lattice vibrations under compression.^55^ The *C*_v_ for NiSc_2_S_4_ and NiSc_2_Se_4_ materials at 300 K and 0 GPa is 314 J mol^−1^ K^−1^ and 330 J mol^−1^ K^−1^, respectively, which corresponds to the mass- and structure-dependent growth of the heat capacity, as was discussed above according to entropy results. The heat capacity at constant pressure *C*_p_ demonstrates an analogous trend when the values are compared in the sequence 0 GPa > 2 GPa > 4 GPa at any temperature (see Fig. 7(d) and 8(d)). This means that the heat capacity of the lattice decreases under pressure because of a strengthening of the interatomic bonds and an increase in the phonon frequencies. Despite the relatively low sensitivity of the heat capacity to pressure change, its effect increases with rising temperature and generally reflects an increase in the lattice rigidity without loss of thermodynamic stability under compression.^56^

The thermal expansion coefficient, α, was measured in order to estimate the change in the volume of NiSc_2_X_4_ spinels during heating at different pressures. According to Fig. 7(e) and 8(e), α shows a high sensitivity to both temperature and pressure; however, the temperature-dependent behavior of α is similar to that of *C*_v_, but α becomes more sensitive to pressure than *C*_v_. In particular, α rapidly increases at low temperatures due to the increasing effect of anharmonic lattice vibrations with rising thermal energy. At higher temperatures, the increase saturates, and α attains a constant value; this happens because the materials tend to reach the classical Dulong–Petit limit.^57^ At 300 K and 0 GPa, the thermal expansion coefficient α equals 6.3 × 10^−5^ K^−1^ in the case of NiSc_2_S_4_ and 6.4 × 10^−5^ K^−1^ in the case of NiSc_2_Se_4_. The slightly higher value of α in selenide is caused by the larger size of the selenium atom and the higher polarizability of selenium compared to sulfur, resulting in lattice softening and more anharmonic vibration and thus in a higher temperature-dependent volume change. The intrinsic lattice softness of the Ni–Se and Sc–Se bonds results in larger thermal expansion of the selenide compared to the sulfide. During hydrostatic compression of both spinels, their thermal expansion coefficients significantly decrease, as expected for condensed matter at high pressure.^58^ This reduction is caused by the decreased amplitude of lattice vibrations and reduced anharmonic interactions between the atoms, and lattice rigidification, which decreases thermal dilatation.

The effect of temperature on the unit cell volume *V* for NiSc_2_X_4_ at various pressures is shown in Fig. 7(f) and 8(f). Regardless of the pressure applied, there is a monotonic increase in *V* with increasing temperature due to the conventional thermal expansion of the crystal structure. The temperature increase causes increased vibration amplitudes of atoms, which results in greater mean interatomic distances and hence an increase in *V*. There is a significant decrease in *V* with increased pressure; this is seen by the following order of volumes for any constant temperature: 0 GPa > 2 GPa > 4 GPa. This shows that any pressure applied to the structure leads to a contraction of the lattice. Contraction of the lattice is a result of shorter interatomic distances and stronger interactions between the atoms due to the pressure. However, there is still an expansion of volume *V* at higher temperatures for all the pressures, but the slope of *V*(*T*) is decreased at higher pressures.

## 4 Conclusion

Mechanical, electronic, magnetic and thermodynamical characteristics of the NiSc_2_X_4_ (X = S and Se) spinels are investigated through the WIEN2k package, and the results highlight their potential applications in spintronics devices. The calculated formation enthalpies, Born stability criteria, absence of any imaginary phonon modes, along with stable AIMD simulations, validate the thermodynamic and structural stability of these spinels. In mechanical studies, the estimated values of the Poisson's ratio and the B_0_/G ratio indicate high ductility over the studied pressure range. From the results of the electronic structure, it is evident that with an increase in pressure, there is a decrease in the direct band gap, and the material shows semi-conducting behavior in the spin-down channel but metallic behavior in the spin-up channel. The magnetic calculations have shown that a total magnetic moment of 2.00 *µ*_B_ is observed for both NiSc_2_X_4_ (X = S and Se) spinels, which originates from the Ni sites, showing robust ferromagnetism. The thermodynamic properties in terms of pressure- and temperature-dependence have been calculated using the quasi-harmonic Debye model. Entropy and Debye temperature are observed to be functions of both temperature and pressure. The entropies at 300 K and 0 GPa are calculated to be 430 J mol^−1^ K^−1^ for NiSc_2_S_4_ and 465 J mol^−1^ K^−1^ for NiSc_2_Se_4_, and their respective Debye temperatures are found to be 320 K and 316 K. These findings, including structural stability, pressure dependent tunable properties and robust ferromagnetism, prove that the NiSc_2_X_4_ spinels can be used for next-generation spintronics/magneto-optoelectronic devices.

## Conflicts of interest

There are no conflicts to declare.

## Data Availability

The corresponding author will provide the data generated during the study upon a reasonable request.

## References

[cit1] Incorvia J. A. C., Xiao T. P., Zogbi N., Naeemi A., Adelmann C., Catthoor F. (2024). *et al.*, Spintronics for achieving system-level energy-efficient logic. Nat. Rev. Electr. Eng..

[cit2] Bai L., Feng W., Liu S., Šmejkal L., Mokrousov Y., Yao Y. (2024). Altermagnetism: Exploring new frontiers in magnetism and spintronics. Adv. Funct. Mater..

[cit3] Zhang L., Liu Y., Wu M., Gao G. (2025). Electric-field- and stacking-tuned antiferromagnetic FeClF bilayer: The coexistence of bipolar magnetic semiconductor and anomalous valley Hall effect. Adv. Funct. Mater..

[cit4] Yahi H., Meddour A. (2017). First principle investigation of structural, electronic and magnetic properties of cubic Cd0.9375TM0.0625S (TM = Ni, Co and Fe). J. Magn. Magn. Mater..

[cit5] Han J., Cheng R., Liu L., Ohno H., Fukami S. (2023). Coherent antiferromagnetic spintronics. Nat. Mater..

[cit6] Wei X. P., Liu X., Sun H. K., Zhang Y. L. (2025). Theoretical investigations of the mechanical, electronic, magnetic and optical properties of Heusler Mn2HfP and Mn2HfSi alloys. Mater. Today Commun..

[cit7] Robail M., Noor N. A., Iqbal M. W., Ullah H., Mahmood A., Naeem M. A., Shin Y. H. (2022). Comprehensive study of ferromagnetic MgNd2X4 (X = S, Se) spinels for spintronic and solar cells device applications. Ceram. Int..

[cit8] Hooch Antink W., Lee S., Lee H. S., Shin H., Yoo T. Y., Ko W. (2024). *et al.*, High-valence metal-driven electronic modulation for boosting oxygen evolution reaction in high-entropy spinel oxide. Adv. Funct. Mater..

[cit9] Zhang L., Liu Y., Xu Z., Gao G. (2023). Electronic phase transition, perpendicular magnetic anisotropy and high Curie temperature in Janus FeClF. 2D Mater..

[cit10] Alizadeh A., Roudgar-Amoli M., Bonyad-Shekalgourabi S.-M., Shariatinia Z., Mahmoudi M., Saadat F. (2022). Dye sensitized solar cells go beyond using perovskite and spinel inorganic materials: A review. Renewable Sustainable Energy Rev..

[cit11] Sundaresan A., Ter-Oganessian N. V. (2021). Magnetoelectric and multiferroic properties of spinels. J. Appl. Phys..

[cit12] Abiodun I. C., Edem M. E., Agbor O. E. (2024). Investigation of the structural and electronic properties of ternary AB2X4-based materials via density functional theory for optoelectronic applications. Commun. Phys. Sci..

[cit13] Bigiani A., Gasparotto A., Andrea Rizzi G., Sada C., Signorini R., Famengo A., Barreca D. (2026). *et al.*, Efficient and selective seawater oxidation by manganese ferrite electrocatalysts obtained via a vapor phase strategy. J. Mater. Chem. A.

[cit14] Alsobhi B. O., Almeshal A. (2023). Structural, elastic, thermodynamic, electronic, magnetic, thermoelectric and optical investigation of chromate spinels TCr2O4 (T = V2+, Mn2+, Fe2+) for optoelectronic applications. Mater. Chem. Phys..

[cit15] Haq M. A. U., Javed M., Mumtaz R., Ullah H., ur Rehman A., Wabaidur S. M. (2024). *et al.*, Mechanical and thermal response of XFe2O4 (X = Zn, Ag and Co) spinel ferrites via IR spectroscopy and first-principles calculations. Phys. Scr..

[cit16] Ak F., Özdemir E. G., Aliabad H. A. R. (2025). Semiconducting character analysis of RhY2O4 oxide spinel via GGA, GGA + mBJ and GGA + U approximations. Indian J. Phys..

[cit17] Özdemir E. G., Balmumcu F. I. (2024). Magnetic, pressure-dependent elastic and band gap calculations of VCo2O4 oxide spinel via GGA, GGA + mBJ and GGA + U. Phys. B.

[cit18] Özdemir E. G., Doğruer S. (2023). Electronic, magnetic and pressure-induced elastic investigations of MnY2O4 oxide spinel. Eur. Phys. J. Plus.

[cit19] Özdemir E. G., Merdan Z., Aliabad H. A. R. (2024). Effects of applied different potentials on electronic and half-metallic characteristics and pressure-dependent elastic and thermodynamic properties of VRu2Br4 spinel. J. Magn. Magn. Mater..

[cit20] Özdemir E. G., Doğruer S. (2023). The structural, magnetic and pressure-induced elastic predictions of ZrPd2O4 oxide spinel via GGA, GGA + mBJ and GGA + U approximations. J. Magn. Magn. Mater..

[cit21] Raza S. A., Mustafa G. M., Ameer M. A., Noor N. A., Farooq Z., Mumtaz S., Moussa I. M. (2025). Investigation of MnSc2X4 (X = S, Se) spinels to unveil their potential for optoelectronic and thermoelectric applications. RSC Adv..

[cit22] Kartal Y. G., Ahmed W. A. A., Özdemir E. G., Doğruer S., Balmumcu F. I., Merdan Z. (2025). An alternative material obtained for spintronic applications using first-principles approximations: TiV2Se4 spinel. Theor. Chem. Acc..

[cit23] Qadoos A., Rashid M., Alkhaldi N. D., Alresheedi N. M., Boukhris I., Mahmood Q., Nasir M. N. (2025). Electronic, magnetic, optical, and thermoelectric properties of rare earth-based CaCe2(S/Se)4 spinels for spintronic and energy harvesting applications. J. Rare Earths.

[cit24] Kattan N. A., Rouf S. A., Alkhaldi H. D., Hassan M., Al-Qaisi S., Aljameel A. I., Mumtaz U. (2025). Study of half metallic ferromagnetism and thermoelectric properties of the spinels MgCo2(S/Se)4 for spintronic and energy harvesting. J. Inorg. Organomet. Polym. Mater..

[cit25] Jamaï I., Ziati M., Bekkioui N., Ez-Zahraouy H. (2024). Structural, electronic, optical, thermoelectric, and thermodynamic properties of XIn2M4 (X = Cd, Zn; M = S, Se, Te) spinels for solar cell and thermoelectric devices: first-principles study. Phys. Scr..

[cit26] Blaha P. , Schwarz K., Madsen G. K. H., Kvasnicka D. and Luitz J., WIEN2k: an Augmented Plane Wave Plus Local Orbitals Program for Calculating Crystal Properties, Vienna University of Technology, Vienna, Austria, 2001

[cit27] Petersen M., Wagner F., Hufnagel L., Scheffler M., Blaha P., Schwarz K. (2000). Improving the efficiency of FP-LAPW calculations. Comput. Phys. Commun..

[cit28] Koller D., Tran F., Blaha P. (2012). Improving the modified Becke–Johnson exchange potential. Phys. Rev. B: Condens. Matter Mater. Phys..

[cit29] Otero-de-la-Roza A., Luaña V. (2011). Gibbs2: A new version of the quasi-harmonic model code. I. Robust treatment of the static data. Comput. Phys. Commun..

[cit30] Noor N. A., Hassan M., Rashid M., Alay-e-Abbas S. M., Laref A. (2018). Systematic study of elastic, electronic, optical and thermoelectric properties of cubic BiBO3 and BiAlO3 compounds at different pressure by using ab initio calculations. Mater. Res. Bull..

[cit31] Mahmood Q., Noor N. A., Jadan M., Addasi J. S., Mahmood A., Ramay S. M. (2020). First-principle investigation of ferromagnetism and thermoelectric characteristics of MgCr2X4 (X = S, Se) spinels. J. Solid State Chem..

[cit32] Alsaiari N. S., Aslam Khan M., Arooj M., Noor N. A., Alomayrah N., Mumtaz S., Al-Buriahi M. S. (2026). Study of thermodynamic, mechanical, photo-catalytic, optoelectronic and thermoelectric properties of ferromagnetic CoSc2 (S/Se)4 spinels for energy conversion devices: DFT+ mBJ calculations. RSC Adv..

[cit33] Chen X., Yi W., Tang G., Yang B., Liu X. (2020). qvasp. Comput. Phys. Commun..

[cit34] Evans D. J., Holian B. L. (1985). The Nose–Hoover thermostat. J. Chem. Phys..

[cit35] Zhang L., Zhao Y., Liu Y., Gao G. (2023). High spin polarization, large perpendicular magnetic anisotropy and room-temperature ferromagnetism by biaxial strain and carrier doping in Janus MnSeTe and MnSTe. Nanoscale.

[cit36] Ali M. A., Naqib S. H. (2020). Recently synthesized (Ti1−xMox)2AlC (0 ≤ x ≤ 0.20) solid solutions: deciphering the structural, electronic, mechanical and thermodynamic properties via ab initio simulations. RSC Adv..

[cit37] Qureshi M. W., Ali M. A., Ma X. (2021). Screen the thermomechanical and optical properties of the new ductile 314 MAX phase boride Zr3CdB4: A DFT insight. J. Alloys Compd..

[cit38] Ali M. A., Hossain M. M., Uddin M. M., Hossain M. A., Islam A. K. M. A., Naqib S. H. (2021). Physical properties of new MAX phase borides M2SB (M = Zr, Hf and Nb) in comparison with conventional MAX phase carbides M2SC (M = Zr, Hf and Nb): Comprehensive insights. J. Mater. Res. Technol..

[cit39] Fan Q., Zhang W., Yun S., Xu J., Song Y. (2018). III-nitride polymorphs: XN (X = Al, Ga, In) in the Pnma phase. Chem.–Eur. J..

[cit40] Zhang W., Chai C., Fan Q., Song Y., Yang Y. (2020). Six novel carbon and silicon allotropes with their potential application in photovoltaic field. J. Phys.: Condens. Matter.

[cit41] Zhandun V. S. (2021). The magnetic, electronic, optical, and structural properties of the AB2O4 (A = Mn, Fe, Co; B = Al, Ga, In) spinels: Ab initio study. J. Magn. Magn. Mater..

[cit42] Maqsood S., Javed M. A., Mumtaz S., Al-Sadoon M. K. (2024). Computational study of Cd-based chalcogenide spinels CdSm2(S/Se)4 for spintronic applications. Chalcogenide Lett..

[cit43] Mahmood Q., Nazir G., Alzahrani J., Kattan N. A., Al-Qaisi S., Albalawi H. (2022). *et al.*, Room temperature ferromagnetism and thermoelectric behavior of calcium based spinel chalcogenides CaZ2S4 (Z = Ti, V, Cr, Fe) for spintronic applications. J. Phys. Chem. Solids.

[cit44] Al-Qhtani M., Mustafa G. M., Mazhar N., Bouzgarrou S., Mahmood Q., Mera A. (2022). *et al.*, Half metallic ferromagnetism and transport properties of zinc chalcogenides ZnX2Se4 (X = Ti, V, Cr) for spintronic applications. Materials.

[cit45] Al-Daraghmeh T. M., Zayed O., Mustafa G. M., Zelai T., Younas B., Albalawi H. (2024). *et al.*, Rare earth based MgPm2X4 (X = S, Se) spinel chalcogenides for spintronic and thermoelectric applications. J. Rare Earths.

[cit46] Raebiger H., Ayuela A., Nieminen R. M. (2004). Intrinsic hole localization mechanism in magnetic semiconductors. J. Phys.: Condens. Matter.

[cit47] Saeed Y., Nazir S., Shaukat A., Reshak A. H. (2010). Ab initio calculations of Co-based diluted magnetic semiconductors Cd1−xCoxX (X = S, Se, Te). J. Magn. Magn. Mater..

[cit48] Elahi I., Alay-e-Abbas S. M., Nazir S., Shaukat A., Tahir M. N. (2019). Evaluation of thermodynamics and p-type ferromagnetism of C, Si and Ge doped ZnX (X = S, Se and Te) semiconductors. J. Magn. Magn. Mater..

[cit49] Hosen A., Dahliah D., Mohammad N. F. A., Mousa A. A., Abu-Jafar M. S. (2025). A computational study on the comparative analysis of tetragonal complex metal hydride Q2FeH5 (Q = Mg, Ca, Sr) for hydrogen storage applications. Int. J. Hydrogen Energy.

[cit50] Gurunani B., Ghosh S., Gupta D. C. (2024). Comprehensive investigation of half-Heusler alloy: Unveiling structural, electronic, magnetic, mechanical, thermodynamic, and transport properties. Intermetallics.

[cit51] Shukla V., Bhatnagar A., Verma S. K., Pandey A. P., Vishwakarma A. K., Srivastava P. (2021). *et al.*, Simultaneous improvement of kinetics and thermodynamics
based on SrF2 and SrF2@Gr additives on hydrogen sorption in MgH2. Mater. Adv..

[cit52] Gurunani B., Gupta D. C. (2025). First-principles investigation of thermoelectric performance in KMnZ (Z = Sn, Pb) half-Heusler alloys. RSC Adv..

[cit53] Johari G. P. (2021). Entropy, enthalpy and volume of perfect crystals at limiting high pressure and the third law of thermodynamics. Thermochim. Acta.

[cit54] Dulong P. L., Petit A. T. (1819). Recherches sur quelques points importans de la theorie de la chaleur. Ann. Chim. Phys..

[cit55] Marx D. and Hutter J., Ab Initio Molecular Dynamics: Basic Theory and Advanced Methods, Cambridge University Press, Cambridge, 2009

[cit56] Mustafa G. M., Younas B., Alkhaldi H. D., Mera A., Alqorashi A. K., Hakami J. (2024). *et al.*, First principle study of physical aspects and hydrogen storage capacity of magnesium-based double perovskite hydrides Mg2XH6 (X = Cr, Mn). Int. J. Hydrogen Energy.

[cit57] Andritsos E. I., Zarkadoula E., Phillips A. E., Dove M. T., Walker C. J., Brazhkin V. V., Trachenko K. (2013). The heat capacity of matter beyond the Dulong–Petit value. J. Phys.: Condens. Matter.

[cit58] Saadi T., Baaziz H., Ghellab T., Latelli H., Telfah A., Charifi Z. (2025). Electronic structure, mechanical and optical properties of hydrogen storage alkaline amides XNH2 (X = Li, Na) compounds. Int. J. Hydrogen Energy.

